# Aesthetic abdominal contouring enhancement with combined abdominoplasty and circumferential liposuction in normal-BMI postpartum Asian women

**DOI:** 10.3389/fsurg.2026.1793884

**Published:** 2026-04-01

**Authors:** Ruomeng Yang, Zhen Song, Jian Wang, Linxi Xia, Hongwei Liang

**Affiliations:** 1 The Department of Plastic Surgery, The Fifth Clinical Medical College of Henan University of Chinese Medicine (Zhengzhou People’s Hospital), Zhengzhou, China; 2 The Department of Plastic Surgery, Henan Provincial People’s Hospital, Zhengzhou, China

**Keywords:** abdominoplasty, body contouring, lipoabdominoplasty, liposuction, surgical techinique

## Abstract

**Backgrounds:**

Abdominoplasty constitutes a principal aesthetic procedure in plastic surgery, with relevance for postpartum women presenting with abdominal wall laxity. Nowadays, an increasing number of patients are undergoing abdominoplasty not merely to address a protruding abdominal appearance, but to pursue a waist-to-hip ratio that approaches an athletic physique.

**Objective:**

This study aims to assess the clinical efficacy of combining abdominoplasty with liposuction within this patient population.

**Methods:**

This retrospective study analyzed 160 postpartum women with BMI (22.1 ± 0.7 kg/m^2^) undergoing combined abdominoplasty and circumferential liposuction. Surgical steps included preoperative marking, tumescent liposuction of flanks, hip rolls, and upper abdomen, followed by abdominoplasty with rectus plication, progressive tension sutures, and meticulous umbilicoplasty. Outcomes were assessed via anthropometric measurements and Visual aid scoring questionnaire.

**Results:**

Waist circumference significantly reduced from 90.9 cm to 80.4 cm postoperatively. Complications included seroma (3 cases), umbilical wound dehiscence (3 cases), and abdominal wound dehiscence (4 cases), all managed successfully. Patient satisfaction significantly improved postoperatively.

**Conclusions:**

Combined abdominoplasty and circumferential liposuction are safe and effective for abdominal contouring, achieving significant waistline refinement and high patient satisfaction. The integrated approach addresses both aesthetic and functional concerns with an acceptable complication profile.

## Introduction

Abdominoplasty has evolved considerably since its introduction in plastic surgery. It is now a well-established and fundamental procedure designed to restore abdominal contour through the excision of redundant skin and adipose tissue, with fascial plication (1–3). Surgical strategies are increasingly individualized based on the extent of cutaneous laxity and the condition of the musculofascial layer (4–6). The integration of liposuction, initially introduced to optimize contouring outcomes (7), has progressively led to the widespread development of the combined technique termed lipoabdominoplasty (8). This integrated approach consistently yields highly satisfactory aesthetic results (9, 10).

Commonly indicated for abdominal protrusion and skin laxity following pregnancy, bariatric surgery, rapid weight loss, or aging (11), abdominoplasty now addresses broader aesthetic expectations. There is a growing desire for surgical outcomes that enhance the overall aesthetic contour of the abdomen, achieve an improved waist-to-hip ratio, and create a fit-appearing physique with features such as “vertical abdominal lines” which refers to the well-defined shape of rectus abdominis muscle and “iliac furrows lines” which refers to the well-defined external oblique muscle bound line. This trend is particularly prominent among Asian women, who often emphasize a slender, well-defined physique.

A growing cohort of patients with normal body mass index now pursue surgery to attain an ideal waist-to-hip ratio and a slender silhouette. This study retrospectively reviewed normal-BMI Asian female patients who underwent combined abdominoplasty and liposuction for abdominal aesthetic enhancement. The surgical technique was adapted to accommodate the specific characteristics of this patient population.

## Methods

A retrospective analysis was performed on postpartum patients receiving abdominoplasty from a single surgeon between February 2022 and April 2025. Inclusion criteria included: (1) dissatisfaction with abdominal contour including skin redundancy, musculofascial laxity, and localized adiposity; (2) age between 18 and 55 years; (3) documented body mass index (BMI) stability (variation <2 kg/m²) for at least six months. Exclusion criteria included: (1) prior history of abdominoplasty; (2) incomplete surgical documentation; (3) comorbidities such as cardiovascular, metabolic, or immune disorders.

This study received approval from the Medical Ethics Committee of The Fifth Clinical Medical College of Henan University of Chinese Medicine (Zhengzhou People's Hospital) and was conducted in accordance with the principles of the Declaration of Helsinki. Written consent was provided, by which the patients agreed to the use and analysis of their data.

## Surgical procedure

### Preoperative assessment and design

**Figure 1:**
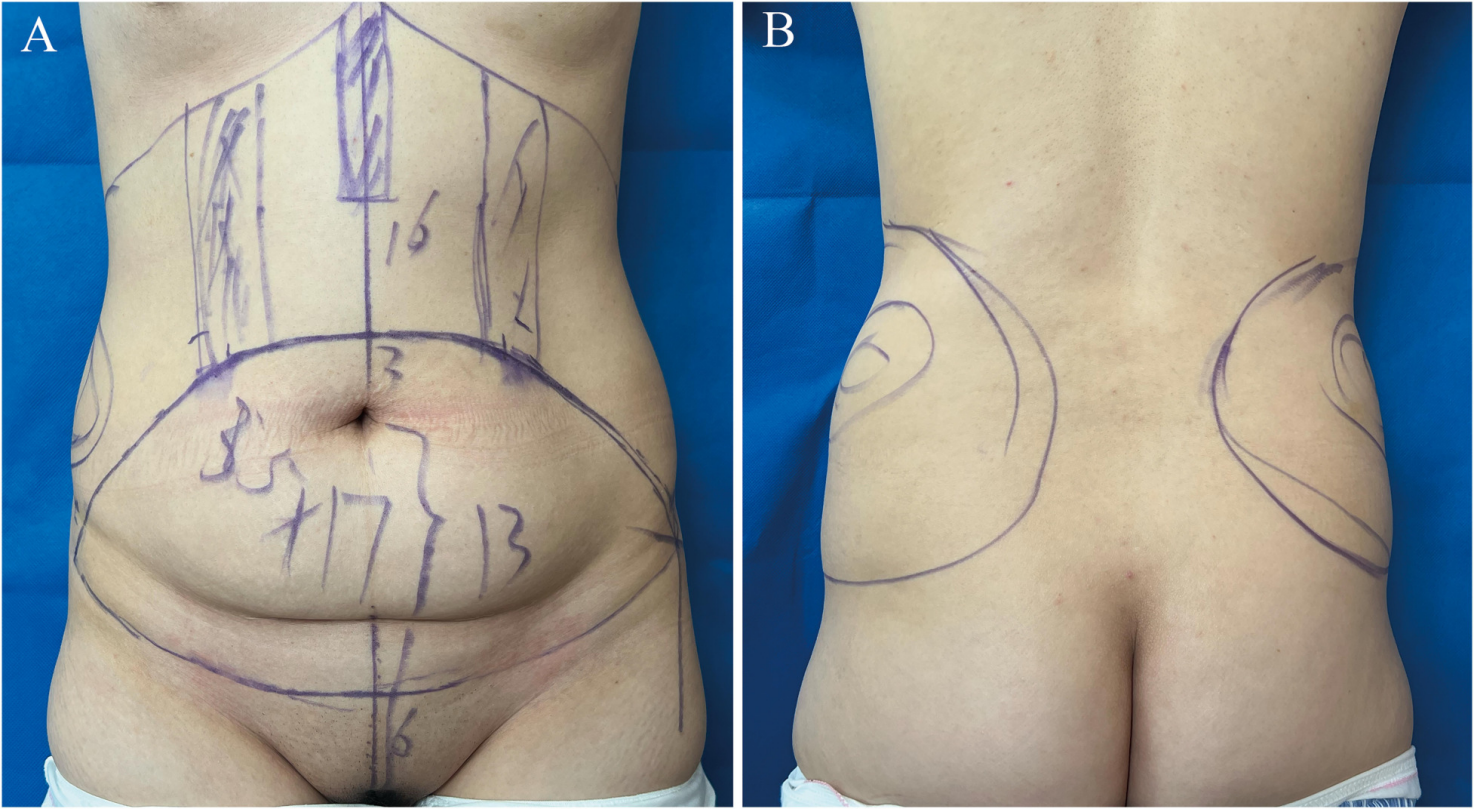
Preoperative design and marking. **(A)** Frontal view of the abdomen illustrating the planned resection and liposuction zones. **(B)** Posterior view demonstrating liposuction areas of the hip rolls and flank regions.

Preoperative evaluation was conducted with patients in a standing position to assess abdominal laxity and protrusion, and adiposity in the abdominal and waist regions. A low transverse incision was designed according to patient preference for scar concealment. The incision length was determined by the amount of redundant skin, which is typically designed medial to the anterior superior iliac spine. If there is excessive skin laxity, the incision may be appropriately extended. The extent of tissue resection and upper incision line were estimates based on closure tension estimated via the pinch test, the final line was defined with a tissue demarcator intraoperatively. The liposuction areas were marked, including flanks, hip rolls, and upper abdomen. Preoperative design and measurement are illustrated in Figure 1.

### Suction-assisted liposuction

**Figure 2:**
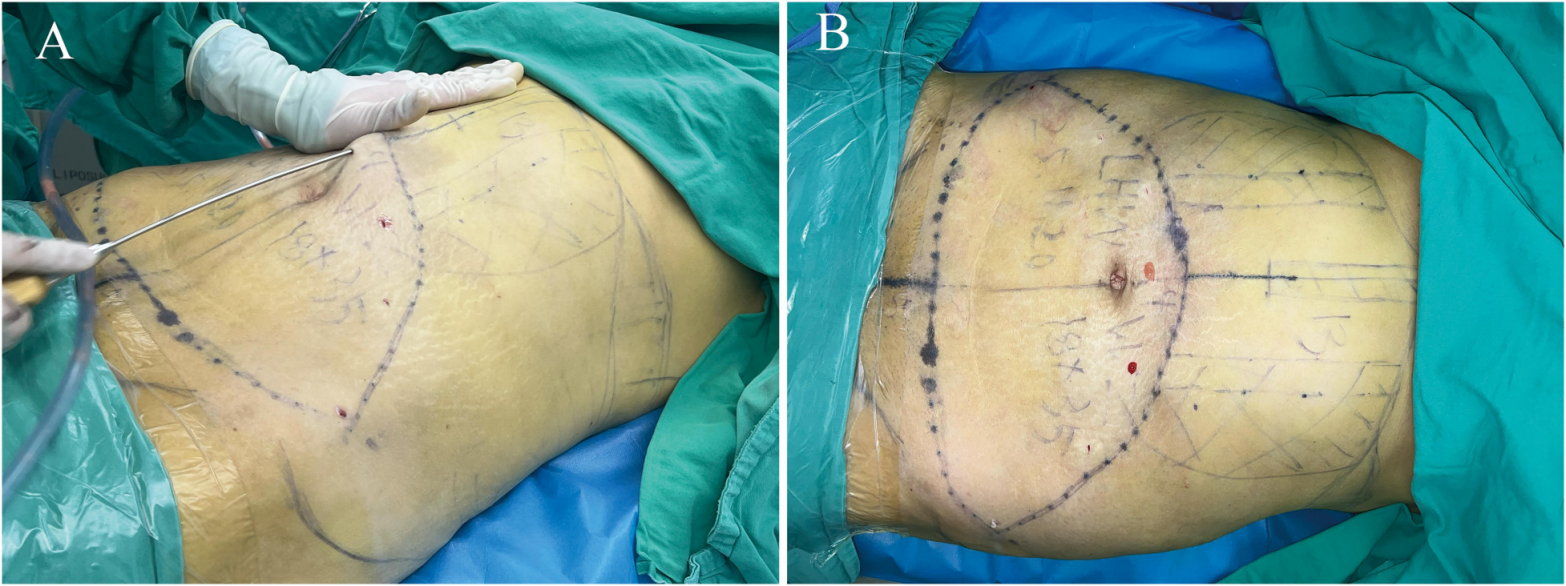
Intraoperative view of extensive liposuction until the subcutaneous adipose layer was significantly thinned.

The patient was placed in the supine position under general anesthesia. The tumescent fluid consisted of 1,000 mL normal saline, 15 mL of 5% lidocaine, 10 mL ropivacaine, and 1 mL epinephrine hydrochloride. Incisions of entry sites were made within the planned resection areas. Tumescent infiltration was administered to both the liposuction zones and the planned abdominal flap resection area. The procedure commenced after a 10 min interval, once the infiltrated tissue attained full turgor, 3 mm blunt tip cannula was usually utilized. Liposuction was initiated bilaterally over the flank and hip areas. To prevent postoperative waistline bulkiness or excessive unevenness with the abdomen, sufficient liposuction of both lateral flank and hip areas is performed until the pinch test confirmed adequate reduction of subcutaneous fat. Finally, liposuction was carried out in the upper abdomen, especially the midline and a parallel vertical strip 5 cm lateral to it for attractive sulcus. Both superficial and deep adipose compartments were completely suctioned. A continuous, flowing, fan-shaped suction technique was employed until a uniformly thin contour was obtained (Figure 2).

### Abdominoplasty

**Figure 3:**
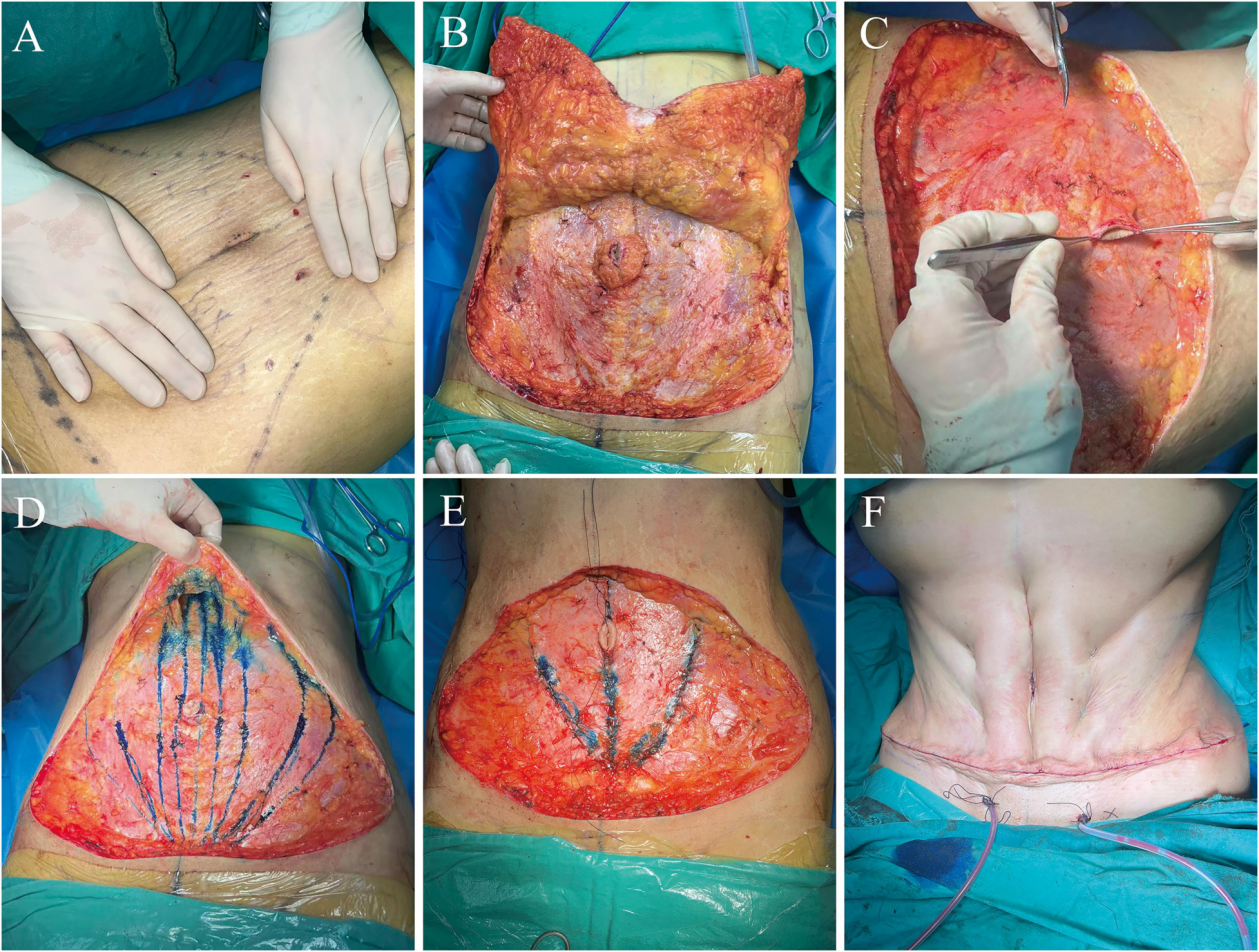
Intraoperative procedures of abdominoplasty. **(A)** Oval umbilical design under skin tension, measuring 2.4 cm in length. **(B)** Sub-Scarpa fascial flap elevation to costal margins with preserved intercostal perforators during xiphoid tunneling. **(C)** Periumbilical skeletonization before plication. **(D)** Marked diastasis recti and plication scope, including lateral rectus borders. The intercostal artery perforators are preserved. **(E)** Symmetrical fascial plication restoring abdominal wall competence with preserved umbilical mobility. **(F)** Progressive tension sutures defining contours of linea alba, linea semilunaris and waistline.

Following complete liposuction of the hip, flank, and upper abdominal regions, the umbilical incision was designed with the skin under tension, measuring approximately 2.4 cm along the vertical axis (Figure 3A). Tumescent infiltration with normal saline was performed circumferentially around the umbilical incision to facilitate dissection and undermining. The umbilicus was circumferentially incised in a near-horizontal plane, detaching it from the surrounding abdominal skin.

A low transverse incision was made, followed by electrocautery-assisted flap elevation under Scarpa's fascia. The abdominal flap was then selectively undermining to the costal margins and xiphoid (Figure 3B). A midline tunnel was carefully dissected in the upper abdomen between the medial borders of the rectus abdominis muscles, ensuring the dissection were not exceeded while meticulously preserving the intercostal artery perforators. Care should also be taken to avoid aggressive upward pulling on the abdominal flap. The umbilical stalk should be skeletonized by removing the surrounding adipose tissue (Figure 3C). The umbilical stalk is secured to the rectus fascia at 12 and 6 o'clock, which are near the dermo-epidermal junction of the umbilicus with non-absorbable sutures. The flap was gently elevated to assess the extent of rectus diastasis, then medial and lateral borders of rectus muscle were marked (Figure 3D). An infraumbilical midline fusiform excision of the Scarpa fascia and underlying adipose tissue exposes the medial borders of the rectus abdominis muscles, enabling myofascial plication. An interrupted mattress suture technique using no. 0 non-absorbable nylon was used for tightening of anterior rectus sheath. The plication knot was buried within the fold. Plication extended superiorly from the xiphoid to the supraumbilical region and inferiorly from the infraumbilical area to the pubic symphysis. In order to further tighten the waist and achieve an ideal waist-to-hip ratio and the “iliac furrows lines” contour, lateral myofascial plication was performed in our study. The specific technique involves marking a horizontal line at the narrowest point of the waist as the superior border for lateral musculoaponeurotic plication. A superior plication width of approximately 3 cm is planned, and a wedge-shaped plication area is marked extending inferomedially. The plication is then performed using 0 non-absorbable sutures. Symmetric plication was performed bilaterally to prevent umbilical displacement (Figure 3E). Significant abdominal wall tautness was achieved following myofascial reinforcement and restoration.

The patient is placed into the semi-Fowler's position. The amount of excess tissue was ultimately assessed under moderate tension using demarcator, then the marked skin and underlying subcutaneous tissue were resected. Under full inferior traction of the abdominal flap, the new umbilical site was marked with methylene blue at its midpoint, followed by a vertical skin incision of 2–2.2 cm. No. 0 Vicryl sutures were applied in a progressive tension suture technique along three vertical rows, including the location of linea alba and linea semilunaris, between the abdominal flap and the muscular wall. Each row contained three to four symmetrically placed stitches. Furthermore, the same suturing technique was applied to the bilateral flanks to enhance the waistline (Figure 3F). Following medial advancement of the superior abdominal flap, the incision was closed in multiple layers to eliminate “dog ear” and accentuate the waistline. Umbilical wound closure employs a two-layer technique: deep dermal inversion with 4–0 Vicryl followed by cutaneous approximation using 6–0 Ethilon.

### Postoperative care

The drains were placed along the bilateral posterior lumbar regions and exteriorized inferiorly through the perineal area inferior to the incision. Intravenous antibiotics and fluid resuscitation were routinely administered during the first two postoperative days. Postoperative care strongly discouraged tobacco use. Patients were advised to maintain adequate hydration with water and liquid nutrition until they could tolerate their regular diet. Thromboprophylaxis measures were consistently implemented. Drains were removed after two consecutive days with output below 30 mL per day. The umbilical sutures were removed on postoperative day 10.

During this phase, patients maintained a flexed trunk posture for three weeks until tissue relaxation allowed full erect standing. An abdominal binder was worn 24 h daily for the initial four postoperative weeks, followed by two months of elastic garment use. Before suture removal at the umbilical incision, silver ion dressings were placed within the umbilicus. From suture removal until one month postoperatively, cotton balls were utilized to maintain shaping and prevent umbilical stenosis.

### Outcome assessment

The evaluation of outcomes was divided into objective measurements and subjective patient assessments. Objective measurements were performed by a surgeon who was not involved in the surgical procedure, with the upper abdominal circumference and lower abdominal circumference measured preoperatively and at six months postoperatively. Subjective assessments were conducted using questionnaires administered preoperatively and at six months postoperatively, evaluated via a visual analog scale (VAS) (0 = dissatisfied, 10 = very satisfied). The questionnaire items included: overall satisfaction with the abdomen, lower abdominal bulge, abdomen flatness, waist definition and body curves, umbilicus shape and position, abdominal striae, scar appearance, impact on self-confidence, and overall score.

## Results

A total of 160 postpartum women were included in this study. The average age was 35.3. The mean BMI was 22.1 ± 0.7 kg/m^2^. The mean liposuction volume was 338.0 ± 72.6 mL, and the mean weight of resected tissue was 474.2 ± 93.0 g. Patients are discharged from the hospital within 5 to 11 days following the surgery. The follow-up period lasted 12.2 ± 2.6 months. Preoperative upper and lower circumferences were 79.6 ± 2.5 cm and 90.9 ± 3.0 cm, respectively. Postoperative measurements obtained at the 6-month follow-up were 77.7 ± 2.3 cm and 80.4 ± 3.1 cm, respectively. The average dimensions of the planned tissue resection were 16.5 cm × 13.0 cm. Measurements were recorded as follows: the distance from the xiphoid to the superior incision margin averaged 17.5 ± 4.5 cm, the distance from the superior incision margin to the umbilicus was 3.5 ± 1.4 cm, and the distance from the inferior incision margin to the fourchette was 5.5 ± 1.0 cm.

**Figure 4:**
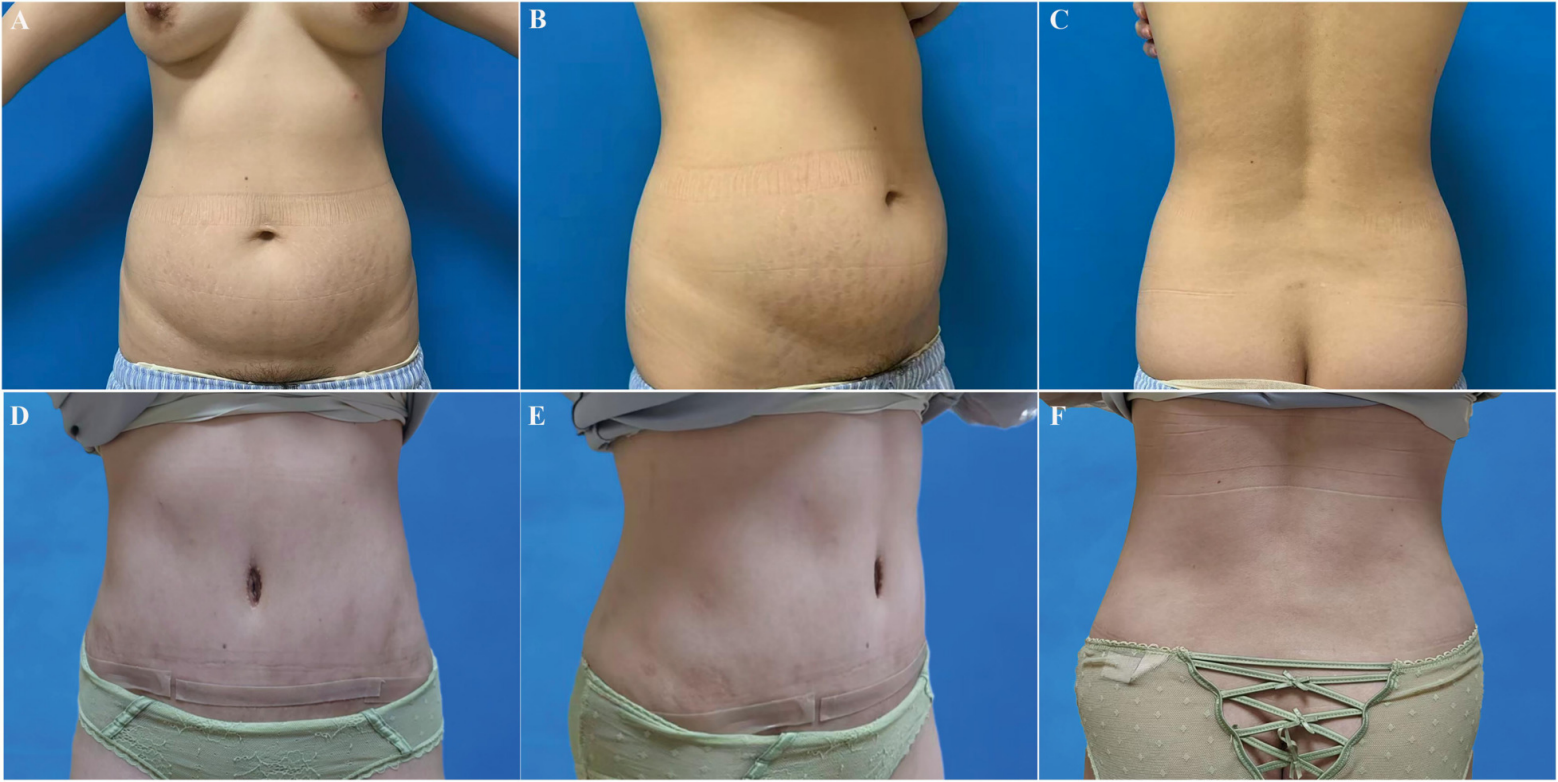
A 31-year-old female with two pregnancies underwent abdominoplasty with liposuction. **(A–C)** preoperative views; **(D–F)** postoperative views.

**Figure 5:**
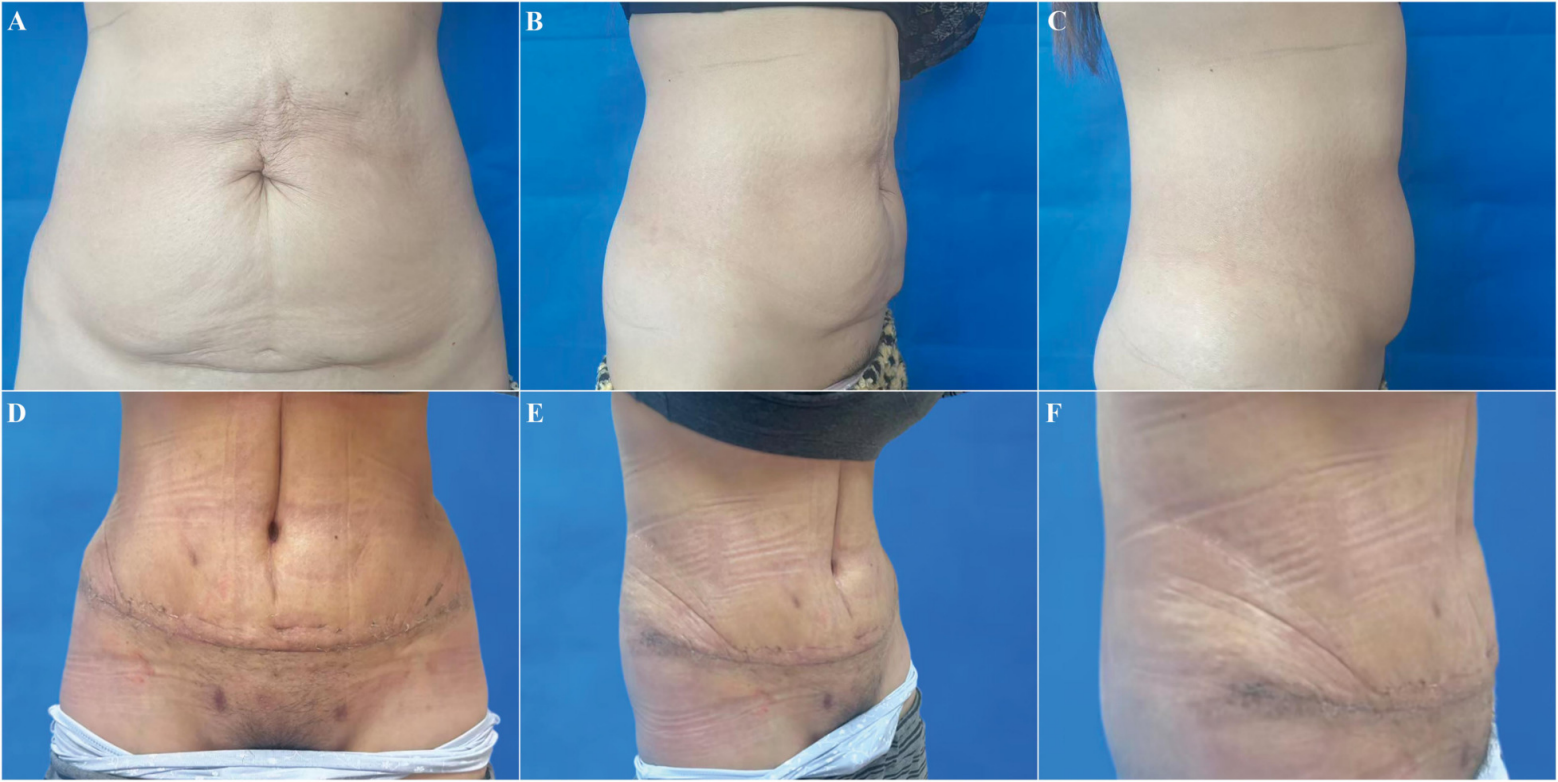
A 40-year-old female with one pregnancy for abdominal wall reconstruction. **(A–C)** preoperative views; **(D–F)** postoperative views.

In the early postoperative period, three patients developed seroma, which resolved successfully after aspiration. Additionally, three patients experienced umbilical wound dehiscence, and four patients had abdominal wound dehiscence. Among these, five cases achieved satisfactory healing through wound care, while the remaining two cases healed successfully following secondary revision. Patients' information was presented in Supplementary Table S1. During follow-up period, patients were satisfied with their abdomen appearance (Figures 4, 5). The VAS score for satisfaction with abdominal morphology increased from 2.25 preoperatively to 7.13 postoperatively (Supplementary Table S2).

## Discussion

The abdominal wall is a principal focus in aesthetic surgery, making abdominoplasty one of the most performed cosmetic procedures worldwide. Postpartum patients frequently exhibit skin laxity, adipose deposition, and rectus diastasis following pregnancy (11, 12). These changes contribute to aesthetic concerns and psychological distress, may also result in functional deficits such as chronic low back pain and core instability. These symptoms can persist even in individuals with normal BMI. Since its introduction in the 19th century, abdominoplasty has undergone continual technical refinements aimed at optimizing aesthetic contours and functional outcomes, while also addressing the complex physical and psychosocial needs of patients. Candidates for abdominoplasty typically demand the following aesthetic outcomes: a flattened and firm abdominal contour, a natural-appearing umbilicus, a well-defined waistline, elimination of striae, and inconspicuous scar.

Abdominoplasty effectively removes redundant skin and performs musculofascial plication, achieving satisfactory aesthetic outcomes. During procedure, flap elevation divides the perforating vessels from superficial inferior epigastric artery and superficial circumflex iliac artery. Additionally, perforators from rectus muscle are also divided to facilitate extensive myofascial plication. This procedure carries a risk of flap ischemia and necrosis, particularly in a triangular area superior to the midline incision, with its apex directed toward the neo-umbilicus (13). Studies indicate that elevation of the skin flap induces hemoconcentration within the microvasculature, promoting microthrombus formation resulting from elevated thrombogenic mediators (14, 15). Conventional abdominoplasty frequently requires wide flap dissection to enable umbilical translocation, potentially increasing complication rates (16). Hence, technical refinements in abdominoplasty have been introduced to enhance safety and optimize results.

In the early 1990s, studies identified safe zones for liposuction in conjunction with abdominoplasty, while strictly prohibiting fat removal from the lateral and central abdominal regions (7, 17). However, performing abdominoplasty and liposuction as separate procedures necessitates a two-stage approach and prolongs recovery to achieve optimal outcomes. In 2001, Saldanha first introduced lipoabdominoplasty by standardizing selective undermining to preserve perforators, safely combining liposuction and abdominoplasty (9). Laser-assisted and power-assisted liposuction are now routinely combined with abdominoplasty to optimize precision, safety, and efficiency (4, 10, 18, 19). Moreover, tumescent infiltration facilitates safe liposuction by achieving tissue exsanguination, vascular compression, and elimination of thrombogenic mediators, thereby optimizing flap viability in abdominoplasty (13). Additionally, patients often present with concerns regarding adiposity in the hip rolls, which disrupts the harmonious truncal silhouette. Our findings indicate that thorough liposuction of both superficial and deep fat layers in the flanks and hip rolls promotes a harmonious lumbar lordosis and accentuates lumbar dimples, thereby refining the waistline contour. Similarly, comprehensive liposuction of the upper abdomen is instrumental in defining anatomical landmarks such as the linea alba and linea semilunaris, which enhances overall abdominal definition. For pubis fullness, mons liposuction is also strongly advised (13, 20).

In this study, the patient cohort primarily consisted of non-obese postpartum women presenting with skin laxity, whose main goal was to achieve an abdominal contour resembling a fitness physique. Therefore, we first performed comprehensive liposuction of the flunk and hip regions to prevent excessive flap thickness. Additionally, during rectus abdominis plication, a wedge-shaped plication of the lateral musculoaponeurotic layer was concurrently performed. This combined approach further reduces the abdominal circumference and, through the plication, helps to create defined lower abdominal oblique grooves, often referred to as the “iliac furrows lines”, which align more closely with the aesthetic preferences of Asian women. This specific technique and its aesthetic outcomes have been infrequently reported in the existing literature.

In abdominoplasty, extensive undermining and liposuction can create dead space, which is associated with seroma formation. It persists as a predominant complication following abdominoplasty, irrespective of concomitant liposuction, with early studies reporting incidence rates of 100% (21, 22). Postoperative patient activity generates shear forces that impede wound healing and promote seroma formation (23). Excessive tension further compromises distal flap perfusion, contributing to scar widening and mons pubis distortion. Progressive tension sutures (PTS) were originally developed as an advancement in fixation technique, systematically scattering tension across the abdominal flap from superficial fascia to deep fascia (24). For decades, substantial evidence supports the efficacy of PTS in reducing those complications (5, 21, 25). Despite evidence supporting drain elimination following progressive tension suture placement, we maintain drain utilization as a safety measure, a practice consistent with prevailing surgical standards (26).

Sutures are typically placed along the midline or medial hemiabdomen (26). In flaps with substantial adipose tissue suture passage becomes technically challenging. To overcome this, we utilize a 20-gauge needle as a guide by inserting it through the flap, threading the absorbable suture through the needle, and then advancing the needle through the dermis. This technique significantly facilitates suture placement and creates a visible cutaneous dimple for precise localization. In our practice, we create grooves resembling natural anatomy by anchoring the rectus fascia to the superficial dermis. PTS effectively creates defined abdominal contours, achieving the linea alba and linea semilunaris that simulate muscular definition on skin, an aesthetic outcome patients request that persists after suture absorption. Beyond the aforementioned suture sites, PTS application extends to bilateral waist regions for dual purposes: eliminating dead space through flap-to-fascia fixation following flank liposuction and enhancing waistline contour definition. To prevent cutaneous erosion, dermal anchoring points should be established within the deep dermal layer, supplemented by regular dressing changes for risk mitigation.

Umbilicoplasty represents a significant process in abdominoplasty, as the umbilicus serves as a critical aesthetic landmark of abdominal wall (27). Multiple factors influence umbilical aesthetics, including location, size, shape, and depth. Most patients desire a naturally appearing umbilicus characterized by small size, vertically elliptical shape, and superior hooding (28), a minority request a linear configuration. Natural umbilical depth and inconspicuous scarring also significantly influence postoperative patient satisfaction. Consequently, these objectives are achieved mainly by securing the umbilical stalk at multiple points and shortening the umbilical island to create depth and prevent umbilical protrusion (29). Technical variations exist in the anchoring depth near the umbilical dermo-epidermal junction (30). Evidence confirms that deeper fixation at the superior position enhances formation of superior hooding, however, which may lead to visible scars (31). We suggest main anchoring at 12 and 6 o'clock, simultaneously leaving proper activity when suturing, which proves to be successful. Moreover, circumferential defatting of surrounding abdominal subcutaneous tissue can create a natural transition between abdomen and umbilical edges.

Although umbilical stalk fixation enhances aesthetic outcomes, its potential impact on perfusion remains controversial. Umbilical necrosis and dehiscence represent rare yet distressing complications following abdominoplasty, particularly when combined with concurrent procedures (32). During procedure, when deep inferior epigastric artery and umbilical perforators are disrupted, the blood supply relies mainly on umbilicus stalk. Furthermore, mechanical compression from rectus plication may compromise blood flow, potentially influence the supply. We employ multiple strategies to optimize umbilical healing and aesthetics: comprehensive tumescent infiltration and near-horizontal incision placement, periumbilical tissue release, appropriate mobility of umbilicus following myofascial plication, umbilical stalk fixation to the fascia, and preventions of umbilicus stenosis by using cotton pack into umbilicus.

In this study, postoperative complications included seroma in three patients, abdominal wound dehiscence in four patients, and umbilical wound dehiscence in three patients. We propose that the primary causes were inadequate immobilization and ineffective compression bandaging in the early postoperative period for some patients. Due to the extensive surgical dissection required for abdominoplasty, the wound surface produces exudate in the early stages; without effective compression, this can readily lead to hematoma formation. Furthermore, in patients seeking a slim abdominal contour, a greater volume of skin is typically resected. This results in relatively higher tension on the postoperative wound closure, making it susceptible to dehiscence if early mobilization is not restricted. To mitigate the risk of poor wound healing caused by fat liquefaction, we recommend excising excess subcutaneous adipose tissue at the wound margins. In recent years, several studies have demonstrated that preserving the Scarpa's fascia in abdominoplasty can enhance postoperative recovery by reducing the volume of drainage, shortening the duration of drain placement, and decreasing the risk of seroma (33, 34). Friedman et al. (35) has demonstrated that preserving Scarpa's fascia during abdominoplasty effectively preserves lymphatic drainage. In the present study, dissection was carried out beneath the Scarpa's fascia, a technique to which our team is more accustomed. We consider that this plane offers a clearer surgical field, facilitates manipulation, and may contribute to reduced operative time. The overall complication rate in our study was within an acceptable range. However, as this study did not include a comparative analysis between preserving and not preserving the Scarpa's fascia, we are unable to assess the impact of this technique on postoperative outcomes and surgical safety. This comparison will be a key focus of our future research.

This study demonstrates that through an integrated surgical protocol-incorporating refined liposuction, precise tissue excision, strategic utilization of PTS, definitive myofascial plication, and meticulous umbilicoplasty-the procedure effectively accomplishes its primary goals: abdominal flattening, waist definition, natural umbilical appearance, and scar minimization. This study has several limitations. First, the retrospective, single-center design, coupled with the absence of a control or comparison group, inherently limits the strength of our conclusions. As all procedures were performed by a single surgeon, the findings may reflect individual surgical expertise and patient selection biases, thus limiting the generalizability of the results to broader clinical settings. Future prospective, multi-center studies with standardized protocols and control groups are warranted to validate these results and to establish evidence-based guidelines. Furthermore, Patients with high body weight or elevated BMI warrant further assessment regarding the effectiveness of the surgical procedure. The absence of long-term outcome assessment restricts evaluation of sustained efficacy and complication profiles. Finally, an in-depth analysis of complications was beyond the scope of this study and represents a valuable direction for future research. Future prospective, multi-center studies with standardized protocols and control groups are warranted to validate these results and to establish evidence-based guidelines.

## Conclusion

The aesthetic objectives of abdominoplasty encompass abdominal flattening, waist definition, umbilical normalization, scar minimization. By integrating abdominoplasty with precise liposuction techniques, surgeons can safely and effectively sculpt harmonious abdominal and dorsal contours while addressing both functional and cosmetic concerns. This comprehensive approach demonstrates consistent efficacy in meeting patient expectations for abdominal restoration.

## Data Availability

The original contributions presented in the study are included in the article/Supplementary Material, further inquiries can be directed to the corresponding author.
