# Supplementary material for: Aesthetic abdominal contouring enhancement with combined abdominoplasty and circumferential liposuction in normal-BMI postpartum Asian women
Source: Front Surg. 2026 Apr 1;13:1793884. doi: 10.3389/fsurg.2026.1793884 (PMC13079155; doi:10.3389/fsurg.2026.1793884)
Supplement: Supplementary file 1 [file Table1.docx]

**TABLE 1 Patient Demographic (n = 160)**

| **Variable** | **Mean ± SD (range)** | **Value (%)** | ***P*** |
| --- | --- | --- | --- |
| Age (years) | 35.3 ± 4.2 |  |  |
| Body mass index (kg/m^2^) | 22.1 ± 0.7 |  |  |
| Smoking history |  | 6 (3.8) |  |
| Reproductive history |  |  |  |
| Single pregnancy |  | 49 (30.6) |  |
| Multiple pregnancies |  | 111 (69.4) |  |
| Cessation history |  | 138 (86.2) |  |
| Upper abdominal circumference (cm) |  |  |  |
| Pre | 79.6 ± 2.5 |  | *P<0.001* |
| Post | 77.7 ± 2.3 |  |  |
| Lower abdominal circumference (cm) |  |  |  |
| Pre | 90.9± 3.0 |  | *P<0.001* |
| Post | 80.4 ± 3.1 |  |  |
| Rectus plication (cm) | 5.3 ±1.1 |  |  |
| Volume of lipoaspirate (mL) | 338.0 ± 72.6 |  |  |
| Thickness of flap (cm) | 3.1 ± 0.8 |  |  |
| Weight of resected tissue (g) | 474.2 ± 93.0 |  |  |
| Operation time (min) | 257.6 ± 49.0 |  |  |
| Blood loss during operation (mL) | 62.8 ± 23.0 |  |  |
| Complications |  |  |  |
| Seroma |  | 3 (1.9) |  |
| Abdominal wound dehiscence |  | 4 (2.5) |  |
| Umbilical wound dehiscence |  | 3 (1.9) |  |
| Follow-up period (m) | 12.2 ± 2.6 |  |  |
