# Supplementary material for: Aesthetic abdominal contouring enhancement with combined abdominoplasty and circumferential liposuction in normal-BMI postpartum Asian women
Source: Front Surg. 2026 Apr 1;13:1793884. doi: 10.3389/fsurg.2026.1793884 (PMC13079155; doi:10.3389/fsurg.2026.1793884)
Supplement: Supplementary file 2 [file Table2.docx]

**TABLE 2 Visual Aid Scoring Questionnaire**

| Question  (0=extremely dissatisfied.  10= totally satisfied) | Mean score (standard deviation) | | *p* |
| --- | --- | --- | --- |
|  | Preoperative | Postoperative |  |
| Overall satisfaction of abdomen | 2.36 (0.95) | 7.65 (1.06) | <0.001 |
| Lower abdominal bulge | 1.88 (1.66) | 7.85 (0.74) | <0.001 |
| Abdomen flatness | 1.95 (0.64) | 7.82 (0.79) | <0.001 |
| Waist definition and body curves | 2.45 (0.78) | 6.87 (0.61) | <0.001 |
| Umbilicus shape and position | 3.23 (0.62) | 6.41 (0.62) | <0.001 |
| Abdominal striae | 2.14 (0.96) | 7.54 (1.02) | <0.001 |
| Scar appearance | - | 4.97 (0.83) | - |
| Impact on self-confidence | 1.63 (0.65) | 8.3 (0.59) | <0.001 |
| Overall score | 2.25 (1.12) | 7.13 (1.31) | <0.001 |
